# Automated Analysis of Sleep Study Parameters Using Signal Processing and Artificial Intelligence

**DOI:** 10.3390/ijerph192013256

**Published:** 2022-10-14

**Authors:** Muhammad Sohaib, Ayesha Ghaffar, Jungpil Shin, Md Junayed Hasan, Muhammad Taseer Suleman

**Affiliations:** 1 Department of Software Engineering, Lahore Garrison University, Lahore 54000, Pakistan; 2 School of Computer Science and Engineering, The University of Aizu, Aizuwakamatsu 965-8580, Fukushima, Japan; 3 National Subsea Centre, Robert Gordon University, Scotland AB10 7AQ, UK; 4 Digital Forensics Research and Service Centre, Lahore Garrison University, Lahore 54000, Pakistan or; 5 Department of Computer Science, School of Systems and Technology, University of Management and Technology Lahore, Lahore 54770, Pakistan

**Keywords:** autoencoders, biomedical signals, deep learning, EEG signals, sleep study, sleep stage classification

## Abstract

An automated sleep stage categorization can readily face noise-contaminated EEG recordings, just as other signal processing applications. Therefore, the denoising of the contaminated signals is inevitable to ensure a reliable analysis of the EEG signals. In this research work, an empirical mode decomposition is used in combination with stacked autoencoders to conduct automatic sleep stage classification with reliable analytical performance. Due to the decomposition of the composite signal into several intrinsic mode functions, empirical mode decomposition offers an effective solution for denoising non-stationary signals such as EEG. Preliminary results showed that through these intrinsic modes, a signal with a high signal-to-noise ratio can be obtained, which can be used for further analysis with confidence. Therefore, later, when statistical features were extracted from the denoised signals and were classified using stacked autoencoders, improved results were obtained for Stage 1, Stage 2, Stage 3, Stage 4, and REM stage EEG signals using this combination.

## 1. Introduction

In the medical field, there are different areas that have been more vastly studied after technological advancement, and these influence the population in both ways: by exploring new things in physiology and diagnosing diseases with more accuracy. Sleep studies are among these fields that are being studied more frequently in the modern era. These studies help doctors better understand the sleep cycle and normal physiology and also help identify and treat a lot of novel sleep disorders. Sleep disorders are related to multiple comorbidities, including hypertension and cardiac diseases. The National Sleep Foundation (NSF) found in a survey that 40% of patients with hypertension, bone aches, heart disease, diabetes, depression, cancer, lung disease, osteoporosis, retention problems, and stroke report disturbed sleep patterns [1]. Among normal individuals, only 10% report some kind of sleep disorder. Sleep disorders also cause cognitive impairment. Sleep disorders may involve a physical change in the duration of sleep, such as reduced total sleep duration or increased time to fall asleep. This change in sleep duration interferes with the normal aging process. The NSF divides sleep disorders into two types, i.e., primary sleep disorders that include sleep-disordered breathing (SDB), sleep-wake disturbances, insomnia, movement disorders (restless leg syndrome (RLS) and periodic limb movement), and secondary sleep disorders that are caused by other diseases such as chronic pain, gastroesophageal reflux, frequent urination, dyspnea, chronic preventable lung disease, or asthma. Primary sleep disorders are diagnosed on the basis of true knowledge of sleep stages and their normal patterns. Mostly all sleep disorders are initially suspected on a clinical basis, but the confirmed diagnosis of the specific disorder is made with the help of polysomnography. Polysomnography (PSG) is an array of physiological signs which are recorded during the whole night when a person is at rest in sleep. These multivariate physiological signs, also known as biosignals, include electroencephalograms (EEG), electrocardiograms (ECG), electrooculograms (EOG), and electromyograms (EMG). Among these, EEG is mostly used by sleep specialists to diagnose sleep-related problems. After overnight recording, these biosignals are interpreted by the sleep specialist doctors, and scoring is completed following the Rechtschaffen and Kales (R & K) rules, which were identified in 1968 and later modified by the American Academy of Sleep Medicine (AASM) [2]. Based on this scoring, sleep is divided into different stages named wakeness (W), non-rapid eye movement (NREM) sleep, and rapid eye movement (REM) sleep. NREM is further divided into four stages (S-1 to S-4) depending on the scoring performed by sleep specialists. Awakeness is the stage of enlivening a person before falling asleep. Stage 1 is characterized by brain action ease and muscle relaxation. Stage 2 is the genuine sleep stage, where eye movements stop, while stage 3 is the resting stage, where a person’s brain sleeps, which as well becomes more profound in stage 4 of NREM. In the REM sleep stage, a person is deeply asleep, but their eyes are moving quickly. Sleep is a naturally happening state of contemplation distinguished by distorted consciousness and calmness of voluntary and sensory muscles, but the human brain is continuously processing during sleep. Tarokh et al. reported cortical advancement in young people utilizing resting electroencephalogram (EEG) signals [3]. The EEG signals vary in different stages of sleep and also show variable patterns in various sleep disorders. Identifying variations in EEG adequacy, recurrence, and wave examples can aid in both the recognition of various sleep stages and the resolution of sleep-related issues.

EEG signals are most commonly used by physicians to represent the brain’s activity during different sleep stages and in the classification of sleep disorders. Four EEG types exist: alpha, beta, delta, and theta. All the waves have specific characteristics and are related to specific sleep stages. In the REM stage, the EEG waves are of mixed frequency, sawtooth pattern, and low amplitude. In NREM stage 1, alpha waves are present with a 2–7 Hz frequency and the highest amplitude. NREM stage 2 is characterized by sleep spindles of frequency 12–14 Hz. Stages 3 and 4 of NREM are deep sleep stages and represent low-frequency waves of 2 Hz on an EEG. Additionally, the pattern of these waves is not periodic, and constant change occurs over periods of hours during the night. During polysomnography, all these waves are recorded overnight and then interpreted in frames of 5–10 s to categorize the sleep in different stages to identify the patient’s sleep quality. Through this discussion, we can understand how complex the method of interpreting the EEG signals in a sleep study is manually by a physician. It takes hours to generate a conclusive report from these EEG signals. We can now surely understand the need for the invention of a consistent and relevant method that can do or at least help the physician analyze the EEG data and make a conclusive report.

In this study, we used artificial intelligence and signal processing to analyze EEG signals from polysomnography to complete the sleep staging process automatically. The main contribution of this study are as follows:

1. The decomposition of complex EEG signals, also known as noise-contaminated signals, as they may contain an abundance of unwanted information into different intrinsic modes so that signal processing at later stages becomes easy.
2. The application of an autoencoder-based deep neural network to extract nonoverlapping reduced feature pools to improve the classification accuracy of the proposed model.
3. The elimination of the use of a separate feature selection step as it can be performed through the proposed autoencoder-based DNN automatically.

## 2. Literature Review

In polysomnography, EEG signals are the most commonly utilized for the sleep staging process by physicians, as well as in previous research using different automated processes [4,5]. The reason behind their usefulness is that these signals can be effortlessly collected with the help of different wearable advanced hard wares, and they comprise valuable data as well. To make EEG data valuable, time [3], recurrence [4], time-recurrence space-based changes [6], and non-straight component extraction techniques [7] are utilized by different analysts at the element extraction phase of E.E.G signals. This trademark of EEG signals also favors further AI calculations.

During the literature review related to this study, we learned from the website “transmitter.ieee.org” that there are different kinds of smart beds, hanging devices, and wristwatches that subjects can wear to monitor all of the sleep-related signals, and sleep scoring can be performed [8]. This is a very important implication of AI in the sleep medicine field because it provides valuable data that can be automatically used to classify sleep stages. In addition, it eliminates the need for the cumbersome procedure of conventional sleep studies that include staying in a sleep lab overnight with multiple wires and electrodes attached to the person undergoing the sleep study procedure [9]. It also gives auto-classification and specific characteristics of different sleep stages to the physician, which saves their time and allows them to simply analyze the data to identify whether a disorder is present or not. Similarly, in another study, Imtiaz et al. [10] proposed a machine-driven computerized scoring for sleep stages. Their results were 82% accurate during training and 79% accurate during the testing phase of their study. Sen et al. [11] conducted a study in which they noted 41 properties of sleep and categorized them into four stages during element extraction and then used high determination techniques to select valuable data from these characteristics. Additionally, the authors in [12] also proposed a framework for characterizing the wake and sleep stages. During component extraction, they divided every EEG report into eight subgroups. Then they used a random forest classifier to organize the obtained data and generate results.

Santaji and Desai [13] proposed a method for sleep stage classification utilizing machine learning techniques to analyze electroencephalogram (EEG) signals over a 10-s time window. The study deployed decision tree, support vector machine, and random forest models that were used to extract and train on statistical characteristics with varying percentages of the testing dataset with the random forest model revealing a 97.8% accuracy score. Bhusal et al. [14] worked to solve the gradient saturation issue introduced by the sigmoid activation function and to enhance the accuracy with which sleep stages may be classified. The suggested system employed a modified orthogonal convolutional neural network and a modified Adam optimization technique. A shorter time to convergence was achieved because of the noise reduction provided by the updated Adam optimizer. Compared to the state-of-the-art approach, ESSC now converges faster and has higher classification accuracy. Tao et al. [15] addressed three issues such as laborious feature extraction, robust feature learning model, and data acquisition through high-quality equipment. They proposed a new feature relearning technique for automated sleep staging based on single-channel electroencephalography (EEG). Yulita et al. utilized a quick convolutional and long short-term memory-based technique for automatic component learning from EEG signals and a softmax classifier for the programmed sleep stage characterization [16]. Similarly, in another study conducted in Aizu-Wakamatsu, Japan, researchers used the EMD technique to decompose EEG signals into different IMFs [17]. These IMFs were then utilized to obtain entropies and statistical moment features in the classification algorithm. Then SVM was used to classify the different types of sleep disorders.

Currently in the field of AI, the entirety of component extraction, determination, and order assignment methods are employed on the obtained data as independent cycles for sleep stage grouping techniques. Ongoing advancements in the AI field have initiated the development of profound structures and the collection of results from them in a more effective manner. Profound learning strategies have recently exhibited their accomplishment in different exploration regions, such as picture acknowledgment, sound preparation, and common language handling. Likewise, profound models have far-reaching applications in the biomedical region as they utilize learning approaches for specific biomedical signals such as EEG, ECG, EMG, and EOG. Profound learning procedures were utilized on many testing themes, for example, the PC-based assessments of ECG information for heart diseases and the discovery of neurological issues utilizing EEG signals. In our study, we are utilizing EEG signals for the identification of sleep stages.

## 3. Material and Methods

### 3.1. Dataset Description

The dataset used in this study to validate the effectiveness of the proposed model was taken from Physionet [18]. The dataset contains polysomnographic EEG recordings of whole-night sleep taken from Fpz-CZ and Pz-Oz electrode locations. According to the 1968 Rechtschaffen and Kales manual, the EEG recordings were manually rated by professionals into Awake, Stage 1, Stage 2, Stage 3, Stage 4, REM, and Movement Time [19,20,21]. For the experiments, as the motive was to identify different stages of sleep, we excluded the movement time pattern. The recordings were obtained from people ranging from 25–101 years of age without any sleep medication. From each patient, two EEG recordings of about 20 h each were obtained during two subsequent day and night periods. These EEG signals were recorded at a sampling frequency of 100 Hz. In the experiment, each EEG recording was divided into 30-s signals to generate enough samples for the study and explore patterns properly. Therefore, after the segmentation process, a total of 367,200 segments were obtained from 153 recordings of approximately 20 h each. Later, the total number of segmented samples was divided into training and test subsets using a 60–40 percent split. The 60–40 data split was considered to check the classification performance of the proposed model on a big proportion of test data. If the model can properly classify a huge amount of test data, then it can be considered a highly generalized network which is also the aim of this study. The details of the dataset is given in Table 1.

### 3.2. Methodology

**Figure 1:**
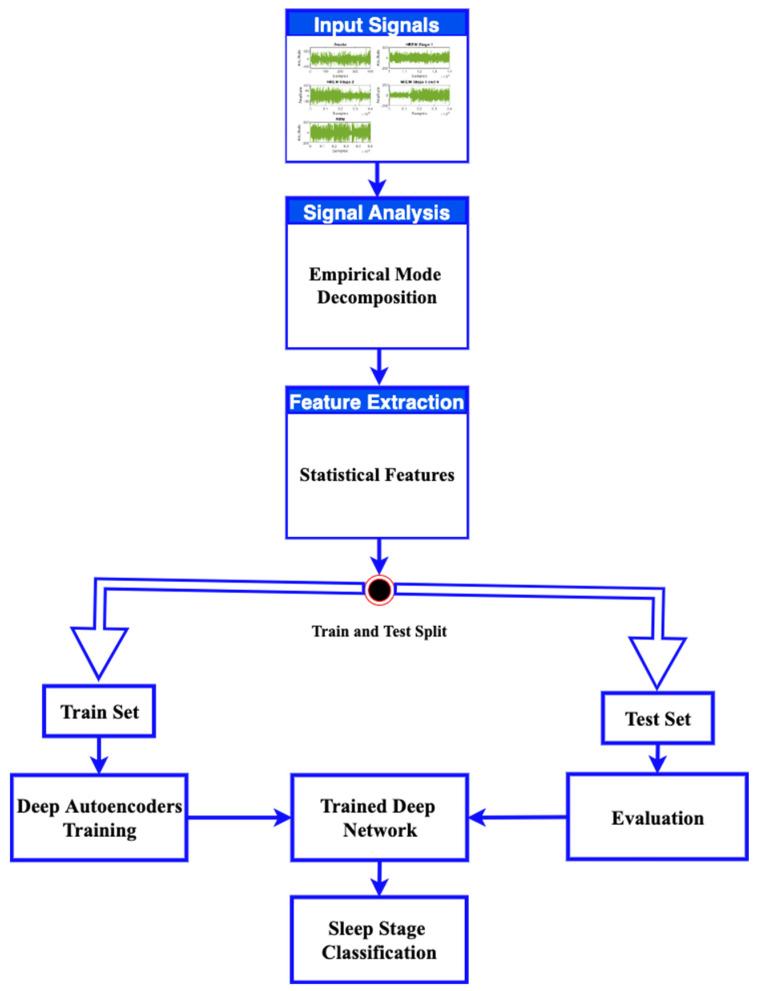
A block diagram of the proposed model.

The current research deals with sleep study staging by using EEG signals. These signals were analyzed by an automated process using signal processing and artificial intelligence, as can be seen in the proposed model given in Figure 1. In this study, we used a dataset that was a record of polysomnographic signals available to the public through the Physionet repository [18]. These recordings were taken during the sleep time of patients presented for the diagnosis or monitoring of different sleep-related symptoms at the Sleep Laboratory in Boston’s Beth Israel Hospital. This database contains 80 hours’ worth of four, six, and seven-channel polysomnographic recordings, including two EEG signals recorded from different brain areas. There are 18 records in this database, and each record comprises four related files.

**Figure 2:**
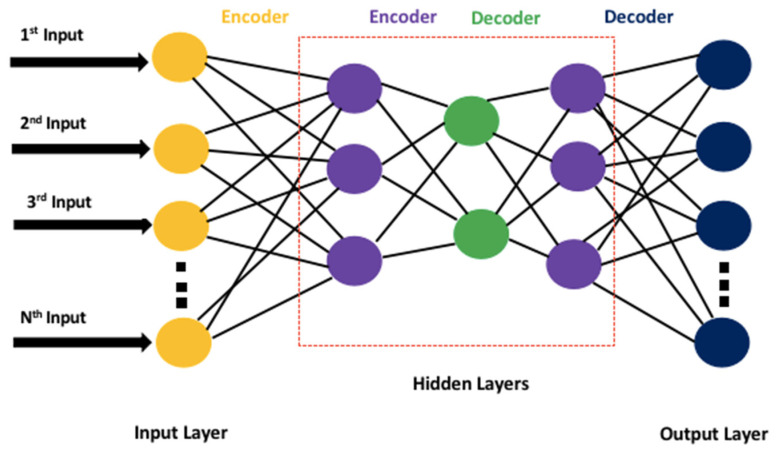
A schematic diagram of stacked autoencoder-based deep neural network.

The obtained data were subjected to empirical mode decomposition (EMD). The EMD analyzes a signal by breaking it down without leaving the time domain. It is mostly used for signals that are natural and non-stationary. It filters out functions that are statistically unrelated to the original signal, which gives an impression of denoising the signal. These functions are known as intrinsic mode functions (IMFs). Obtaining IMFs from real-world signals is important because natural processes such as sleep have multiple events, and each of these events happens at specific time intervals. The decomposition of signals into subcomponents is helpful to analyze signals more easily and efficiently. For instance, feature extraction from each intrinsic mode function through a stacked autoencoder-based DNN is more meaningful as it can result in the generation of non-overlapping features. This type of data is evident in an EMD analysis but quite hidden in the Fourier domain or wavelet coefficients. For this reason, we preferred EMD for collecting useful data from EEG signals. The statistical features were extracted from each of the IMFs and were later provided to the stacked autoencoder-based DNN to classify the sleep stages into awake, REM, and NREM. The proposed scheme for our study is given as follows. The architecture of the stacked autoencoder-based DNN is shown in Figure 2. The DNN was trained using a k-fold cross-validation procedure to minimize the model bias and variance by setting the k = 10. The train and test sets contained a total of six classes, i.e., Awake, NREM Stage 1, NREM Stage 2, NREM Stage 3, NREM Stage 4, and REM. Each class has the data of all the subjects involved in the sleep study during the data recording provided by [18]. The autoencoder-based deep neural network was trained on each class containing the data of all the participants. Therefore, it was subjected to an independent training process.

### 3.3. Evaluation Matrices

To note the efficacy of the proposed model, five performance evaluation criteria were used in this study. These evaluation criteria were class-wise classification accuracy, average classification accuracy, and k-fold cross-validation. The following formulation represents the classification accuracy.

True Positive + True Negative/Total Number of Samples(1)

## 4. Results Analysis

**Figure 3:**
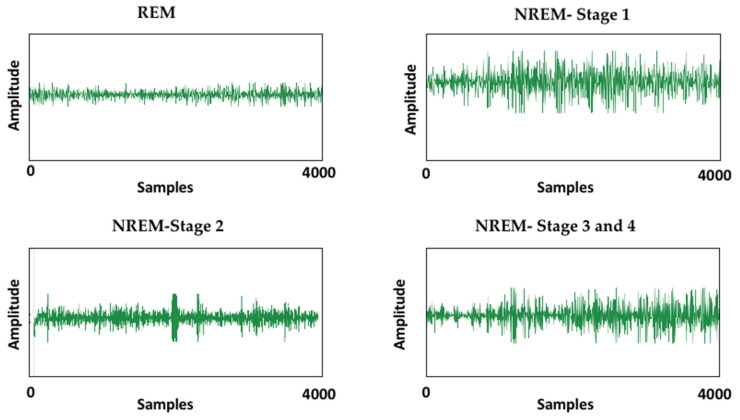
The EEG signals associated with the different sleep stages of a normal human being.

**Figure 4:**
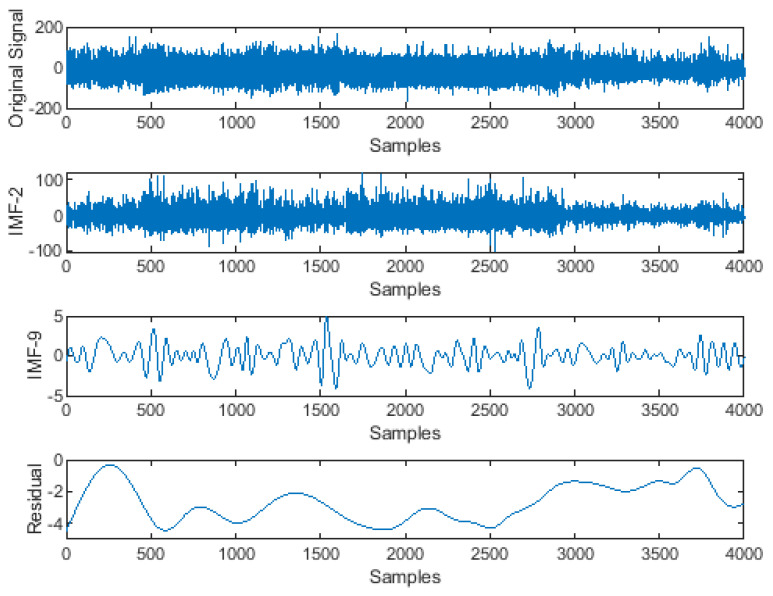
A diagram of an original EEG signal, its different IMFs, and the residual signal after decomposition through the empirical mode decomposition method.

**Figure 5:**
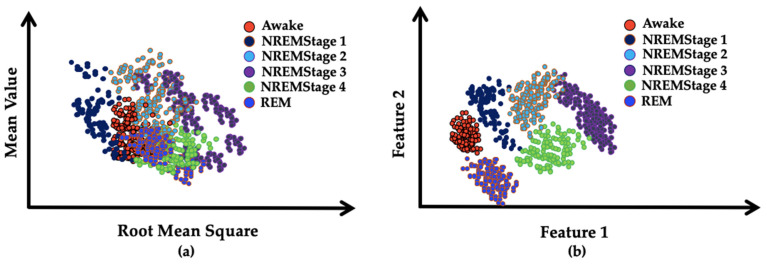
(**a**) Distribution of statistical features without passing though stacked autoencoder-based deep neural network, (**b**) abstract features learned by stacked autoencoders for different classes.

**Figure 6:**
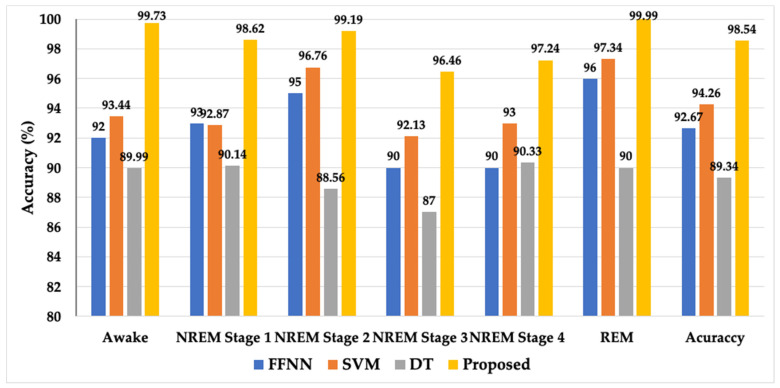
Classification performance of the proposed algorithm and other popular classifiers.

A block diagram of the proposed model is presented in Figure 1. It consists of the division of the EEG recordings into equal-length segments, signal denoising through EMD, feature extraction, and feature reduction and classification steps through stacked autoencoders. In the first step, each EEG recording was divided into 30-s segments. In the next step, the segmented samples were passed through the signal-denoising step through EMD. The denoising of the signal was carried out to remove the effects of artifacts from the contaminated EEG signals. After the denoising of the signals, the statistical parameters mentioned in Table 2 were calculated from the signals. These extracted features were used as descriptors of the original EEG signals related to different classes. Later, these extracted features were provided to a deep neural network created with the help of autoencoders to extract a reduced pool of abstract features and classify the data. In Figure 3, EEG signals related to different sleep stages are shown. As illustrated in the figure, each signal has unique characteristics that can only be understood by a domain expert. These unique characteristics are considered weak descriptors as they can be easily buried under noise. Therefore, to enable automatic classification of these EEG signals into the respective sleep or awake categories, first, these signals are required to be denoised. For this reason, the EEM-based denoising criterion was adopted in this work. As EMD is an adaptive process for signals analysis, it decomposes the input EEG signals contaminated with artifacts into different oscillation scales. These different oscillation scales are termed intrinsic mode functions or modes. For the current study, the EEG signals were decomposed into 10 IMFs, i.e., IMF1 to IMF10. Afterward, the residue signal could not be decomposed further. An original EEG signal and its different IMFs, i.e., IMF 1, IMF2, IMF 9, and its residual, are represented in Figure 4. After the signal decomposition into IMFs, statistical features were extracted from each IMF and passed to a deep neural network formed through stacked autoencoders. The stacked autoencoders were able to learn a reduced set of abstract features, which were used as the final pool of features to complete the classification task. A scattered graph of abstract feature pools can be seen in Figure 5. In this figure, two scatter plots are presented in (a) and (b). The first plot represents an example where two statistical features were plotted directly without the extraction of abstract features through the stacked autoencoder-based DNN. The same trend was observed for the rest of the statistical pairs. It is evident that there is a lot of feature overlap in the feature space. Therefore, when a classifier is trained and tested using such an overlapped feature pool, the classification performance of the classifier deteriorates automatically. To mitigate the issue, in this work, feature 1 and feature 2 represent the first and second vectors of the principal components extracted by the autoencoders, respectively. It is evident from the figure that the features associated with different types of EEG signals form almost distinct clusters, i.e., overlapping among different types of features is minimal. Therefore, when such distinct features were provided to the softmax classifier, the overall classification performance of the stacked autoencoder-based DNN was significantly enhanced, which can be verified in Figure 6. In this figure, the classification accuracy of the proposed model, along with other popular algorithms, is given. These algorithms include a feed-forward neural network (FFNN), support vector machines (SVM), and decision trees (DT). It can be observed that the proposed model achieved the highest level of classification accuracy as compared to other power classification algorithms. These results demonstrate that the proposed algorithm successfully overcame the problem of signal contamination and categorized the data into their respective classes correctly.

To further consolidate the efficacy of the proposed model, the results of the proposed models are compared with those of the state-of-the-art published studies. The comparison is given in Table 3. From this comparison, the proposed model outperformed other techniques. Based on the comparison results shown in Figure 6 and Table 3, it is safe to say that the proposed model can reliably identify the signals related to the different sleep stages of a normal human, even if the signals are complicated by artefacts.

## 5. Conclusions

Identifying a patient’s sleep stages through EEG signals is a cumbersome task, as these signals can easily be contaminated through artifacts. Therefore, in this study, an attempt has been made to design a strategy through which a patient’s sleep stages can be easily identified with high accuracy, even if EEG signals with artifacts are used. The key aspects of this study are presented below:

1. This study presents a mechanism to easily denoise a contaminated EEG signal so that it can be effectively utilized for further analysis and complete the sleep stage classification task.
2. In traditional data-driven classification tasks, a separate feature selection step is required after feature engineering and extraction. It is an important step because it eliminates redundant data from the inputs, hence, reducing the dimension of the data. So, the use of a separate feature selection algorithm in the classification pipeline is considered overhead. It not just increases the time complexity of an algorithm but also enhances the implementation and debugging complexity of the algorithm. Therefore, in this work, a specialized deep neural network based on autoencoders was used for automatic input compression that learned a reduced pool of abstract features. Apart from the dimensionality reduction of the data, the same deep neural network could be used to classify the data. Therefore, this algorithm eliminates the need for separate feature selection and classification steps. Moreover, the abstract feature pool significantly enhanced the classification performance of the deep neural network as the features that belonged to different classes had distinct distribution patterns that reduced the overlapping of the features to a minimalistic level.
3. This study also highlights the need for a non-redundant and non-overlapping feature pool in classification tasks. As it is evident from the results that using features with overlapping patterns deteriorates the final classification accuracy of a classifier. On the other hand, a non-redundant feature pool with distinct distribution patterns that do not overlap each other significantly enhances the performance. The effect of the features as selected by the feature selection algorithm on performance is found to be more positive and significant when compared to the use of all features.
4. The current research study focused on a single parameter, i.e., EEG signals. However, this methodology can be applied to multimodal data. For this purpose, a tweaking of the current model will be required according to the data used. Moreover, this study relied on statistical features. The values of these features can abruptly change with a change in the characteristics of the signals. The future prospects of this work could be the introduction of a sophisticated pattern recognition mechanism to eliminate the dependency on the statistical features set and a more generalized network that can be directly adopted by multimodal data.

## Figures and Tables

**Table 1:** The dataset description.

No. of EEG Recordings (20 Hours Each)	Period of Segmentation (Seconds)	Total Number of Samples after Segmentation	No. of Samples in Train and Test Subsets after Splitting	
			Train (60%)	Test (40%)
153	30	367,200	220,320	146,880

**Table 2:** The statistical features used in this study.

Features	Equations	Features	Equations	Features	Equations
Mean value	$\bar{x}=\frac{1}{N}\sum_{i=1}^{N}x_{i}$	Standard deviation	$\sigma^{2}=\frac{1}{N-1}\sum_{i=1}^{N}{\left(x_{i}-\bar{x}\right)}^{2}$	Root mean square (RMS)	$RMS={\left(\frac{1}{N}\sum_{i=1}^{N}x_{i}^{2}\right)}^{\frac{1}{2}}$
Peak-to-peak value (PPV)	$PPV=\max(x_{i})-\min(x_{i})$	Skewness value (SV)	$SV=\frac{1}{N}\sum_{i=1}^{N}{\left(\frac{x_{i}-\bar{x}}{\sigma }\right)}^{3}$	Margin factor (MF)	$MF=\frac{\max(\left|x_{i}\right|)}{{\left(\frac{1}{N}\sum_{i=1}^{N}\sqrt{\left|x_{i}\right|}\right)}^{2}}$
Crest factor	$MF=\frac{\max(\left|x_{i}\right|)}{(\frac{1}{N}\sum_{i=1}^{N}x_{i}^{2})\frac{1}{2}}$	Impulse factor	$IF=\frac{\max(\left|x_{i}\right|)}{\frac{1}{N}\sum_{i=1}^{N}\left|x_{i}\right|}$	Square root of the magnitude (SRM)	$SRM=(\frac{1}{N}\sum_{i=1}^{N}\sqrt{\left|x_{i}\right|}{)}^{2}$
Kurtosis value (KV)	$KV=\frac{1}{N}\sum_{i=1}^{N}{\left(\frac{x_{i}-\bar{x}}{\sigma }\right)}^{4}$	Kurtosis factor	$KF=\frac{\frac{1}{N}\sum_{i=1}^{N}{\left(\frac{x_{i}-\bar{x}}{\sigma }\right)}^{4}}{(\frac{1}{N}\sum_{1}^{N}x_{i}^{2}{)}^{2}}$		

**Table 3:** The comparison results of the proposed model with other state-of-the-art studies.

Algorithm	RPMs	Average Accuracy
Ahnaf et al. [20]		95.31
Rashedul et al. [17]	1722, 1748, 1772, 1797	94.12
Proposed	1722, 1748, 1772, 1797	98.75

## Data Availability

The data of this case study is publicly available.
